# Swirling Flow and Wall Shear: Evaluating the BioMimics 3D Helical Centerline Stent for the Femoropopliteal Segment

**DOI:** 10.1155/2018/9795174

**Published:** 2018-02-26

**Authors:** Timothy M. Sullivan, Thomas Zeller, Masato Nakamura, Colin G. Caro, Michael Lichtenberg

**Affiliations:** ^1^Vascular/Endovascular Surgery, Minneapolis Heart Institute at Abbott Northwestern Hospital, Minneapolis, MN, USA; ^2^Department of Angiology, Universitäts-Herzzentrum Freiburg Bad Krozingen, Bad Krozingen, Germany; ^3^Division of Cardiovascular Medicine, Toho University, Ohashi Medical Center, Tokyo, Japan; ^4^Department of Bioengineering, Imperial College, London, UK; ^5^Vascular Center Klinikum Arnsberg, Arnsberg, Germany

## Abstract

The BioMimics 3D self-expanding nitinol stent represents a strategy for femoropopliteal intervention that is alternative or complementary to deployment of drug-coated stents or balloons. Whereas conventional straight stents reduce arterial curvature and disturb blood flow, creating areas of low wall shear, where neointimal hyperplasia predominantly develops, the helical centerline geometry of the BioMimics 3D maintains or imparts arterial curvature, promotes laminar swirling blood flow, and elevates wall shear to protect against atherosclerosis and restenosis. In the multicenter randomized MIMICS trial, treatment of femoropopliteal disease with the BioMimics 3D (*n* = 50) significantly improved 2-year primary patency (log-rank test *p* = 0.05) versus a control straight stent (*n* = 26), with no cases of clinically driven target lesion revascularization between 12 and 24 months (log-rank test *p* = 0.03 versus controls). In geometric X-ray analysis, the BioMimics stent was significantly more effective in imparting a helical shape even when the arterial segment was moderately to severely calcified. Computational fluid dynamics analysis showed that average wall shear was significantly higher with the helical centerline stent (1.13 ± 0.13 Pa versus 1.06 ± 0.12 Pa, *p* = 0.05). A 271-patient multicenter international MIMICS-2 trial and a 500-patient real-world MIMICS-3D registry are underway.

## 1. Introduction

Peripheral artery disease (PAD) is one of the most prevalent, morbid, and mortal diseases worldwide, affecting more than 202 million individuals [1]. Between 2000 and 2010, the prevalence of PAD grew at a rate of 13.1% in high-income countries and 28.7% in low- and middle-income countries [1]. Patients with PAD have an increased risk of myocardial infarction, stroke, and death, as well as significant quality of life (QOL) impairment. Initial treatment of PAD in patients with intermittent claudication is directed at lifestyle and behavior modification along with medical management in order to slow the disease and symptom progression. When these methods fail to provide symptomatic relief, revascularization is appropriate by endovascular or surgical means, as recommended by current society guidelines [2].

**Figure 1:**
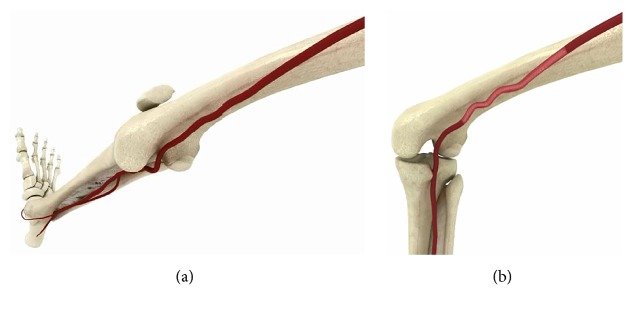
(a) Gentle curvature of the superficial femoral artery (SFA) with the leg extended. (b) With the knee flexed, the distal SFA adopts a helical pattern to accommodate vessel slack.

The femoropopliteal arterial segment is the most common anatomic location of occlusive PAD [3]. The superficial femoral artery (SFA) descends along the anteromedial part of the thigh in the femoral triangle before entering and passing through the adductor canal and becoming the popliteal artery, which runs through the knee in close proximity to the joint capsule. The femoropopliteal segment is thus uniquely subjected throughout its length to complex external mechanical stresses, including flexion, compression, and torsion, which may contribute to poor treatment outcomes such as mechanical failure of stents and arterial kinking [3–6]. Atherosclerotic occlusive lesions in the femoropopliteal segment, due to associated blood flow patterns and disturbances and consequent areas of low wall shear, can be long and can involve significant calcification and/or fibrosis [7–9]. The clinical presentation of patients with femoropopliteal lesions can range from asymptomatic or with minor symptoms to QOL-limiting intermittent claudication (IC) or critical limb ischemia (CLI). While, with the leg extended, the SFA has a gentle open spiral shape, during thigh contraction and knee flexion, compression is increased as the distal SFA segment traverses the adductor canal and adopts a more helical pattern (Figure 1).

As endovascular procedures entail lower periprocedural risks and decreased initial costs in comparison with open surgical repair in PAD patients with multiple comorbidities [10], current guidelines recommend an initial endovascular approach for treatment of most types of femoropopliteal disease [11]. The objective in endovascular treatment of PAD is the restoration of patency and blood flow in stenosed or occluded arterial segments, in such a way that restenosis or reocclusion can be avoided along with the need for reinterventions to correct for the failure of primary patency. When primary patency fails, PAD symptoms often recur, providing clinical indications for target lesion revascularization (TLR) or target vessel revascularization (TVR) procedures, which are costly, clinically more risky, and less likely to be successful [12–14]. The causes of low patency rates following endovascular repair of femoropopliteal lesions can include immediate elastic recoil of the treated segment; intimal dissection; late negative vessel remodeling (fibrosis of the adventitia); and an inflammatory process following balloon barotrauma and stent implantation leading to development of neointimal hyperplasia (NIH) and in turn to restenosis [4]. Elastic recoil and intimal dissection can be managed relatively successfully by prolonged balloon dilatation and/or mechanical scaffolding with stents [4, 6]. With the unsatisfactory outcomes following standalone percutaneous transluminal angioplasty (PTA) in many lesion types (1-year vessel patency < 30% [15]) and use of nondedicated bare-metal stents [16, 17], the development of further strategies is ongoing to effectively prevent or limit the activation of the NIH cascade and consequent vessel renarrowing.

The specific endovascular options that have been successively developed in the effort to prevent restenosis in the femoropopliteal segment include dedicated self-expanding nitinol stents, drug-eluting stents (DES), and drug-coated balloons (DCBs). In a recent analysis of femoropopliteal outcomes in clinical trials of different endovascular modalities, updated from the systematic review presented in the Cardiovascular and Interventional Radiological Society of Europe (CIRSE) Standards of Practice guideline on the superficial femoral and popliteal arteries, rates of technical success were uniformly high for endovascular treatment of stenoses (ranges between 98% and 100%) and occlusions (ranges between 81% and 94%). However, 1-year rates of primary patency ranged from 57% (95% confidence interval [CI]: 42%–72%) for PTA, to 66% (95% CI: 67%–91%) for conventional nitinol stents, to 81% (95% CI: 67%–91%) for DCBs and 83% (95% CI: 77%–90%) for DES [4, 18]. The extent to which these systematic review findings represent treatment of real-world patients is not clear, as the 1-year patency rates were not discriminated on the basis of symptoms (IC versus CLI), comorbidities such as renal disease and diabetes, or lesion characteristics (baseline lesion length, percentage of stenosis, or presence and degree of calcification) [18]. Clinical trials generally enroll patients with shorter rather than longer lesions (lesion lengths ranged from 5 to only 10 cm in the DES trials represented in the systematic review [4]), fewer rather than more occluded lesions, and only mildly to moderately calcified lesions. Clinical trials likewise tend to exclude patients with Rutherford class 5 and 6 disease (the most severe clinical presentations), and they do not generally involve head-to-head comparisons of related interventional modalities.

In real-world practice, for example, when DCBs are employed as primary treatment of femoropopliteal disease, complementary stent deployment is planned or at least reserved for treatment of suboptimal results. The pivotal DCB trials focused on a closely defined set of lesions arising from relatively uncomplicated PAD that would not require complementary stenting; they explicitly excluded severe calcification and an inability to completely predilate the lesion; and the use of SFA stents was not permitted [19–23]. Consequently, the rates of bailout stenting were only between 2.5% and 7.0% in the pivotal DCB trials, whereas the rates of complementary stenting were between 28.8% and 35.5% (with the rate of stenting related to lesion length and degree of occlusion) when use of the same DCBs was analyzed in registry studies. In the IN.PACT Global registry, the rate of complementary stenting was 53% when lesion length exceeded 25 cm. The pharmacokinetic profile of DCBs, while varying with the type of excipient coating employed, is distinct from that of DES in terms of drug-release and tissue-distribution characteristics but still also time limited (with the drug eluted by DCBs remaining at therapeutic levels for less than 1 year), whereas loss of patency in the SFA due to restenosis can occur as late as 3 to 4 years after treatment [24]. That reality suggests a role for complementary deployment of a stent associated with durable outcomes in cases when DCBs are employed as primary treatments.

## 2. The BioMimics 3D Helical Centerline Stent

An alternative or complementary strategy for limiting the NIH cascade and restenosis associated with endovascular interventions in the femoropopliteal segment focuses on imparting a helical shape to the stented arterial segment and thereby inducing a laminar swirling flow of blood that increases mixing within the blood and generates an antirestenotic and atheroprotective elevation in wall shear. That is the strategy behind development of the BioMimics 3D helical centerline stent system (Veryan Medical, Horsham, UK). The design of the BioMimics 3D builds on the principles underlying the most recent generation of nitinol stents dedicated for use in the femoropopliteal arterial segment—radial support, flexibility, durability, clarity of visualization, and accuracy of delivery—and adds three-dimensional helical centerline geometry, for the purpose of providing biomechanical stability and also generating swirling flow within the stented segment.

This self-expanding nitinol stent is under evaluation in 3 separate multicenter prospective clinical trials in the MIMICS Clinical Trial Program (Table 1). (1) The multicenter MIMICS study, the first-ever randomized trial comparing two differently designed bare-metal nitinol stents (in most nitinol stent studies, the investigational device is compared with PTA) [25]; (2) the prospective MIMICS-2 investigational device exemption (IDE) study, which is now evaluating the helical centerline stent in 271 patients at 43 sites in 3 different countries (the United States, Japan, and Germany); and (3) the prospective observational MIMICS-3D registry, which is evaluating the helical centerline stent in a real-world clinical population, with targeted enrollment of more than 500 patients and with a dedicated subgroup analysis of device performance as a complementary treatment in procedures involving DCBs are included. Clinical results of the randomized MIMICS study, which have been published out to 24 months, demonstrate that differences in stent design do influence clinical outcomes in the femoropopliteal segment [25].

The current review considers the evidence supporting the use of the BioMimics 3D helical centerline stent as an effective alternative or complementary strategy for limiting NIH in the femoropopliteal segment. To contextualize this strategy, the review begins with a summary of what is known about the relationship between wall shear and restenosis.

### 2.1. Wall Shear, Restenosis, and the Effect of Swirling Flow

**Figure 2:**
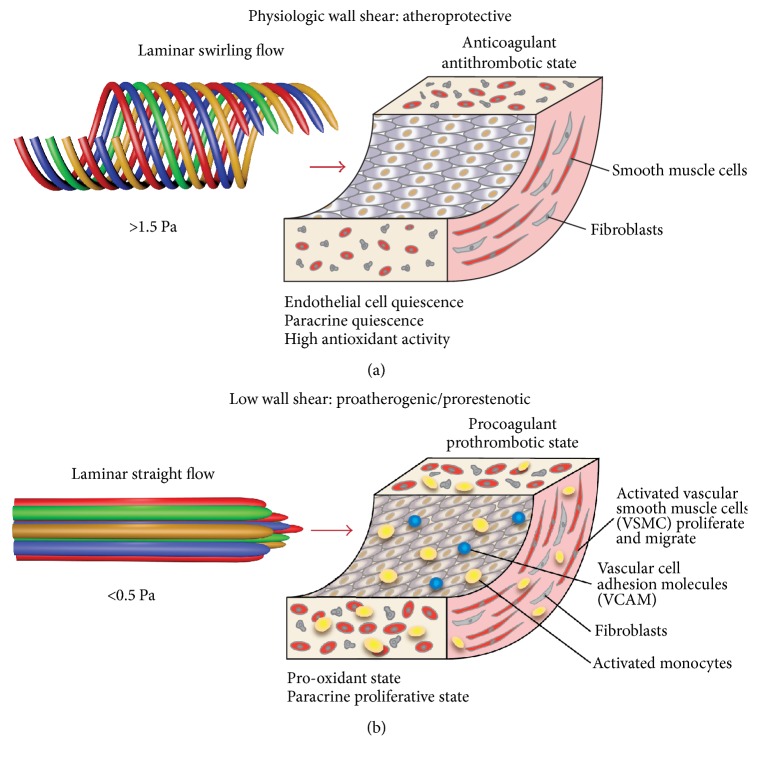
A model of atherogenesis/restenosis, showing differences caused by physiologic arterial wall shear (a) and low (proatherogenic/prorestenotic) wall shear (b) in upregulation of endothelial-cell genes and proteins that are atheroprotective/antirestenotic or atherogenic/restenotic. Adapted from Malek et al. with permission [30].

It has long been understood that disturbances in arterial fluid mechanics contribute to atherogenesis [26]. Arterial geometry is commonly helical, causing the blood to adopt a laminar swirling flow pattern, with the higher velocity of flow occurring toward the arterial wall rather than in the center of the vessel as in the case of arteries that are straight [27]. The naturally occurring laminar swirling flow increases mixing within the blood, elevates the wall shear on endothelial cells, and promotes the diffusion of oxygen to the arterial wall, protecting against the development of atherosclerosis and restenosis [28, 29]. The vessel endothelium is not a passive nonthrombogenic surface but rather a dynamically responsive vascular element, which produces autocrine and paracrine factors under the functional regulation of local hemodynamic forces [30]. A critical determinant of endothelial function and phenotype [30–32], wall shear is the tangential component of frictional force generated at the vessel wall by the flow of blood. High (normal or physiological) wall shear induces endothelial-cell (EC) quiescence and an* atheroprotective *gene-expression profile, whereas low shear stress stimulates an* atherogenic* phenotype (Figure 2) [30]. That is to say that atherosclerosis and NIH mainly occur at locations where wall shear is low [7, 8, 26]—wall shear > 1.5 Pa being atheroprotective, while wall shear < 0.5 Pa is related to the development of atherosclerosis and restenosis.

As the SFA is a long, relatively straight vessel, it is exposed to low wall shear under resting conditions [9]—a circumstance that predisposes it to atherosclerotic disease while also serving as a factor that confounds the healing process after endovascular injury. Implantation into the SFA of a stent with a straight cylindrical configuration will act to further straighten the vessel, so that while the stent may restore vessel patency, it can alter effective arterial flexibility and reduce the capability of the vessel to shorten naturally (as in an unstented state), opening the way for kinking and buckling of the vessel at the ends of the stented segment as well as for possible stent fracture [5]. The introduction of a straight stent may jeopardize the antirestenotic swirling blood flow commonly imparted by the natural helical arterial geometry. The implantation of a stent with a helical centerline can avoid this possibility by imparting swirling flow to the diseased segment of the SFA requiring intervention.

### 2.2. Device Design

**Figure 3:**
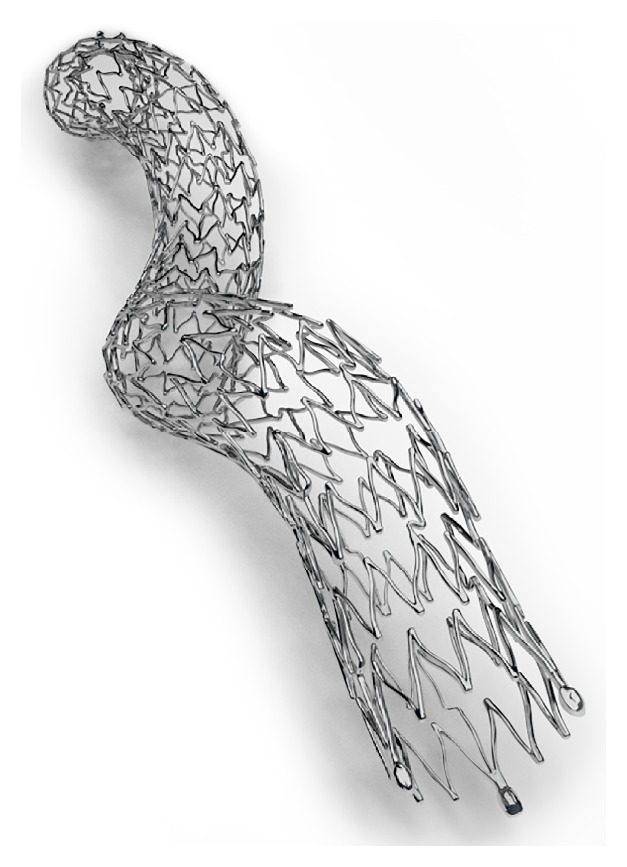
The BioMimics 3D stent (Veryan Medical, Horsham, United Kingdom).

The BioMimics 3D helical centerline stent was designed to impart a helical shape to vessel morphology and thereby induce a laminar swirling flow that will elevate antirestenotic and atheroprotective wall shear (Figure 3). The strut pattern of the helical centerline stent promotes high device flexibility while still retaining sufficient stiffness to impart a helical shape to the stented segment even when it is moderately to severely calcified. The helical centerline curvature of the stent is stored within the shape memory of the nitinol alloy. Patterns of short and long connectors between the strut crowns support the helical geometry and flexibility of the stent. The device design includes transition zones consisting of the last three crowns at each end of the stent. In these transition zones, crowns are increased in length, to reduce the outward radial force of the end of the stent on the vessel wall and to avoid any flow disturbances that might arise due to a step-change between the stent and proximal or distal vessel segments. To avoid the edge restenosis that may be caused by disturbed flow patterns at junctures between stents and normal vessel segments [33], the ends of the helical centerline stent are formed to be collinear with the normal vessel to ensure optimal blood flow into and from the stent.

The BioMimics 3D self-expanding helical centerline stent received Conformité Européene (CE) marking in November 2012. The stent is indicated to improve luminal diameter in the treatment of symptomatic de novo and restenotic lesions up to 140 mm in length in native superficial femoral and/or proximal popliteal arteries with reference vessel diameters ranging from 3.5 mm to 6.0 mm.

## 3. Evaluations to Date of the Helical Centerline Stent

### 3.1. Animal Study in Porcine Common Carotid Arteries

**Figure 4:**
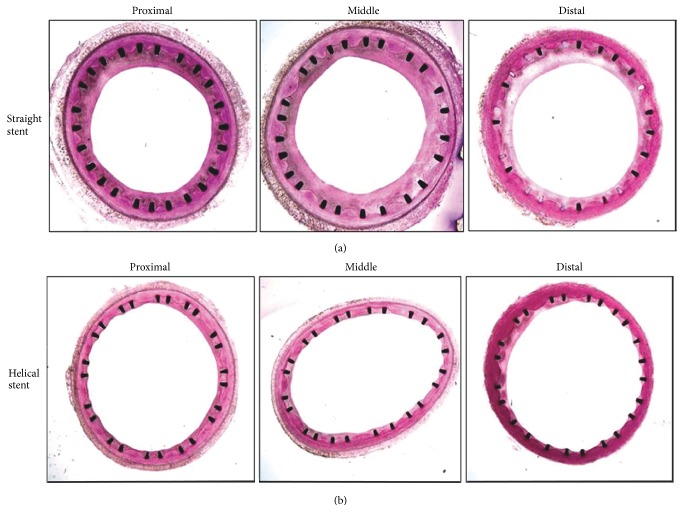
Transverse sections of porcine common carotid arteries treated with (a) a control straight stent and (b) the helical centerline stent at 1 month after stent deployment. From Caro et al. with permission [28].

In a preclinical study assessing the effect of the helical centerline stent on the development of NIH, the device was compared with a control straight nitinol stent in one or the other of the common carotid arteries of 10 healthy pigs [28]. Digital subtraction angiography, using a thin filament of contrast, was performed immediately after stent deployment and indicated the presence of a swirling blood flow pattern in the helical stent but not in the control straight stent. Transverse Doppler ultrasound, using a technique described by How et al. [34], also demonstrated the presence of swirling flow in the helical stent. At animal sacrifice 1 month after deployment, histology revealed significantly less NIH in vessels implanted with the helical centerline stent than in vessels implanted with the control straight stent (*p* < 0.01). Mean neointimal thickness was reduced by an average of 45% in vessels implanted with the helical centerline stent compared with vessels implanted with the control straight stent (0.2 ± 0.09 mm^2^ versus 0.37 ± 0.08 mm^2^, *p* < 0.05). Luminal cross-sectional area was significantly greater with the helical centerline stent than with the control straight stent, as can be seen in Figure 4.

Both stents used in this animal study changed the morphology of the common carotid arteries of healthy pigs. However, by imparting a helical curvature to the treated segment, the helical centerline stent distinctly generated a swirling flow of blood. The geometrical and flow changes persisted up to the time of sacrifice at 4 weeks [28]. Swirling flow, as discussed, is associated with a high level of wall shear, which has been found to limit NIH activation and appears to be responsible for the improved histological outcomes with the helical centerline stent in this study [35].

### 3.2. The Randomized Controlled MIMICS Trial (NCT02163863).

In the prospective, multicenter, controlled MIMICS trial, 76 patients with TASC (Trans-Atlantic Inter-Societal Consensus) II A and B SFA lesions were randomized 2 : 1 to receive the helical centerline stent (*n* = 50) or a control straight nitinol stent (LifeStent, CR Bard, Phoenix, AZ) (*n* = 26). One patient receiving the helical centerline stent had a TASC II C lesion. Ultrasound, angiography, and X-ray imaging review were performed by an independent core laboratory. Eight centers participated in the study, and follow-up continued for 24 months. The primary safety endpoint—freedom from a composite of all-cause mortality, index limb amputation, and TLR through 30 days—was based on an objective performance goal (OPG) of 88% set by the VIVA Physicians [15]. The primary efficacy endpoint, 6-month freedom from clinically driven TLR (CDTLR), was based on an OPG of 67% deriving from a review of recent literature [36–38]. The secondary endpoint of primary patency was defined as freedom from >50% stenosis identified by formal angiography or duplex ultrasound. Loss of primary stent patency in a treated vessel segment was defined as an increase in the peak systolic velocity ratio of >2.0 or the occurrence of CDTLR.

Zeller et al. have published clinical results for the MIMICS trial out to 24 months [25]. The helical centerline stent achieved the primary efficacy and safety endpoints. At 1 year, the Kaplan-Meier survival estimate for primary patency was 80% for patients who received the helical centerline stent versus 71% for patients who received the control straight stent. At 2 years, the survival estimate for primary patency was 72% versus 55%, respectively, a statistically significant primary patency advantage for the helical centerline stent (log-rank *p* = 0.05).

**Figure 5:**
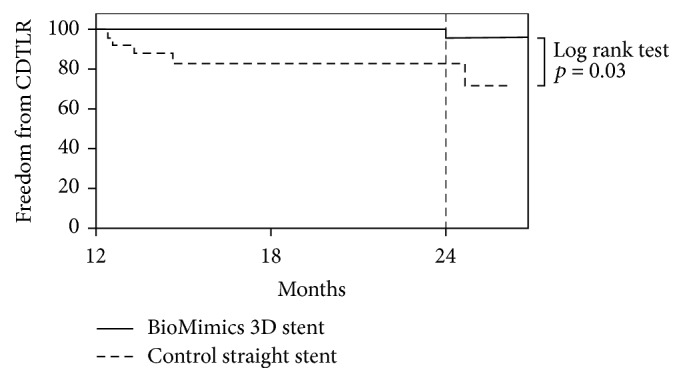
Kaplan-Meier curves of freedom from clinically driven target lesion revascularization (CDTLR) after independent event adjudication for the helical centerline stent versus the control straight stent for the landmark period between 12 and 24 months in the MIMICS clinical trial.

At 1 year, the Kaplan-Meier survival estimate for freedom from CDTLR was 91% for patients who received the helical centerline stent versus 92% for patients who received the control straight stent. At 2 years, notably, freedom from CDTLR remained 91% for patients who received the helical centerline stent while being reduced to 76% for patients who received the control straight stent. Due to the relatively small sample size, the difference in freedom from CDTLR at 2 years was not statistically significant (log-rank test *p* = 0.14). However, for the period between 12 and 24 months for patients implanted with the helical centerline stent, there were no additional cases of CDTLR, whereas, for patients implanted with the control straight stent, there was a threefold increase in the cases of CDTLR (from 8% at 12 months to 24% at 24 months). For this landmark analysis of the period from 12 months to 24 months, there was a statistically significant advantage for the helical centerline stent in freedom from CDTLR (log-rank test *p* = 0.03) (Figure 5).

A post hoc analysis of the MIMICS trial data was conducted to support understanding of how the BioMimics 3D stent performed with regard to a range of patient risk factors, including grade of calcification, percentage of occlusion, number of patent run-off vessels, diabetes status, and lesion length (80 mm cut-off). For patients implanted with the helical centerline stent, the analysis indicated that there was no significant difference in 24-month primary patency related to any of these variables. There was no reduction in the level of curvature in the implanted stent when patients with no calcification or mild calcification were compared versus those with moderate or severe calcification (*p* = 0.14). Comparing patients with lesion length greater than or less than 80 mm, there was no significant difference in 24-month primary patency for the helical centerline stent (73% versus 72%, *p* = 0.98), while the patency difference was significant for the control straight stent (*p* = 0.03).

### 3.3. Helical Curvature, Swirling Flow, and Wall Shear in the MIMCS Trial

Two post hoc geometric analyses, based on X-ray data obtained from all patients in the MIMICS trial, have explored whether the helical centerline stent was able to maintain a higher degree of helical arterial curvature than the control straight stent and then whether stent-modified vessel geometry was associated with a higher level of wall shear due to swirling flow.

During the MIMICS trial, all patients underwent extended-leg and bent-knee X-rays in both anterior/posterior and lateral projections at 1, 6, 12, and 24 months. The original purpose of such detailed X-ray evaluation was to inspect devices for evidence of stent fracture (there were no fractures), but the imaging data also provided a basis for computing the three-dimensional helical curvature of each implanted stent. The stent centerline curvature was calculated using a previously described method [39]. Coordinate points on the centerline of the stented vessel were extracted at regular intervals to form the basis for the centerline curvature calculations, which were performed using a MATLAB program (Mathworks, Natick, MA).

**Figure 6:**
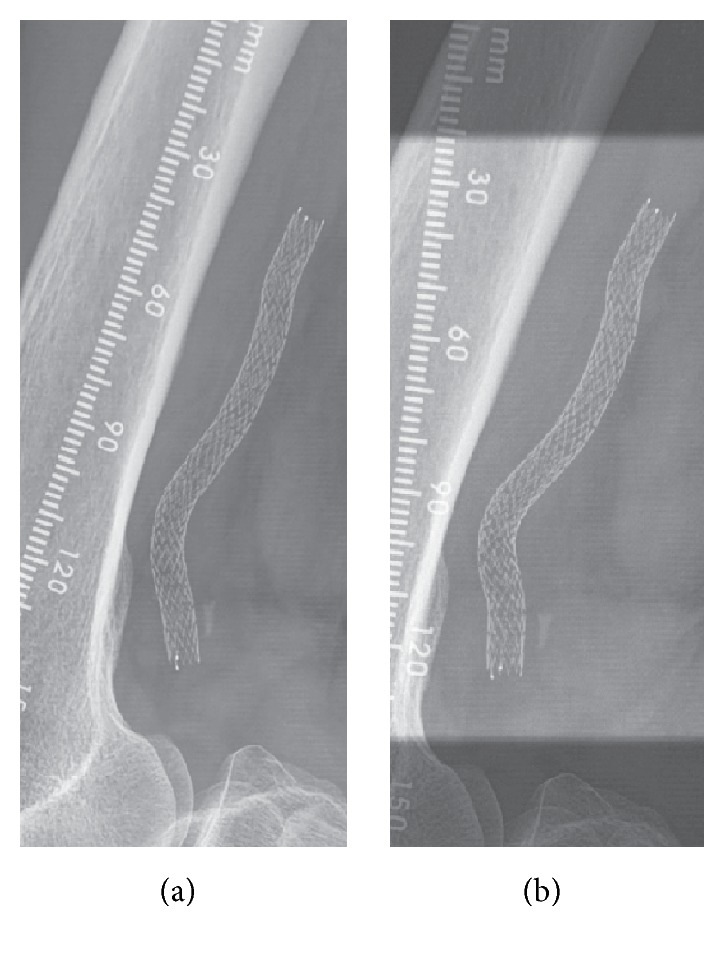
X-ray images of a BioMimics 3D stent in a femoropopliteal location in a patient with knee bent at 90 degrees, at 1 month (a) and 6 months (b) after implantation, showing sustained stent curvature over time.

The analysis revealed that significantly more stent centerline curvature was present in the extended-leg and bent-knee positions of patients who received the helical centerline stent compared with patients who received the control straight stent. For the extended-leg position, the mean stent centerline curvature was 0.0167 ± 0.007 mm^−1^ with the helical centerline stent versus 0.0133 ± 0.004 mm^−1^ with the control straight stent (*p* = 0.019). For the bent-knee position, the mean stent curvature was 0.0198  ±  0.008 mm^−1^ with the helical centerline stent versus 0.0152 ± 0.006 mm^−1^ with the control straight stent (*p* = 0.018). These results in patients from the MIMICS trial confirmed the previous observation from the porcine carotid artery study—that the helical centerline stent imparts a higher level of nonplanar arterial curvature than does a control straight stent, which may be flexible but is not capable of* imparting* curvature that is not already present in the vessel. Also corroborating and extending the findings of the animal study, qualitative analysis of X-ray data over multiple follow-up visits demonstrated that the helical centerline of the BioMimics 3D stent was maintained over time (Figure 6), providing the basis—through the phenomena of swirling flow and high wall shear—for the significant patency and revascularization outcomes through 24 months in the trial.

The reconstructed stent geometry data for the bent-knee position were then used for a computational fluid dynamics analysis of the flow characteristics within the lesion segments in order to calculate average wall shear. In this analysis, three-dimensional flow fields were predicted for each stented vessel segment based on patient-specific duplex ultrasound recordings of physiological flow rates, and an engineering simulation suite (ANSYS 14.0, Ansys, Pittsburgh, PA) computed Navier-Stokes equations. In patients treated in the MIMICS trial, the average wall shear as calculated in this analysis was significantly higher within the helical centerline stent than with the control straight stent (1.13  ±  0.13 Pa versus 1.06 ± 0.12 Pa, *p* = 0.05).

## 4. Conclusion

Arterial geometry is commonly helical, causing the blood to adopt a laminar swirling flow pattern that increases mixing within the blood and elevates the wall shear on endothelial cells, in turn promoting the diffusion of oxygen to the arterial wall and protecting against the development of atherosclerosis and restenosis [28, 29]. Due to its length and relative straightness, the SFA is exposed to low wall shear under resting conditions and is thus predisposed to atherosclerotic disease and to a confounding of the healing process after the injury associated with endovascular intervention. Implantation into the SFA of a stent with a straight cylindrical configuration will act to further straighten the vessel, rendering it inflexible and potentially jeopardizing the atheroprotective swirling blood flow commonly imparted by the natural helical arterial geometry and leading to activation of the NIH cascade. The BioMimics 3D helical centerline stent was designed to impart a helical shape to vessel morphology and thereby induce a laminar swirling flow that will promote antirestenotic and atheroprotective wall shear. In the MIMICS trial, patients with occlusive SFA lesions were randomized to treatment with either the helical centerline stent or a control straight stent. At 24 months, there was a significant improvement in primary patency for the helical stent compared with the control straight stent. There were no occurrences of CDTLR in the helical centerline stent arm between 12 and 24 months after implantation [25].

The MIMICS trial confirmed that differences in the design of stents for deployment in the femoropopliteal artery are relevant to clinical outcomes. The geometric analysis of X-ray image data for patients in the MIMICS trial found a significantly higher degree of helical curvature with the BioMimics 3D stent than with the control straight stent in both extended-leg and bent-knee configurations, and the imparted curvature was shown to be maintained over time. In a post hoc analysis of the device performance with regard to a range of patient risk factors, there was no reduction in the level of curvature in the implanted stent when patients with no calcification or mild calcification were compared versus those with moderate or severe calcification. The computational fluid dynamics modeling of duplex ultrasound data and bent-knee X-ray measurements indicated a significantly higher level of wall shear with the helical centerline stent than with the control straight stent. It is reasonable, then, to infer that both the higher helical curvature and the higher wall shear associated with the helical centerline stent limited the volume of intervention-activated NIH in stented segments in comparison with the control straight stent. This conclusion is supported by the histological findings in the preclinical study in porcine common carotid arteries [28]. To the end of limiting the volume of NIH activated after endovascular intervention and hence the rate of restenosis, the strategy of attending to the phenomena of swirling flow and wall shear with implantation of the helical centerline stent represents a promising alternative to deployment of DES and a potential complement to the use of DCBs. It remains for the results of the MIMICS trial to be confirmed in the larger MIMICS-2 prospective registry and the MIMICS-3D real-world registry (Table 1).

The MIMICS-3D real-world registry will include an important subgroup analysis of the performance of the helical centerline stent when deployed as a complementary treatment in procedures involving DCBs. The likelihood of there being an important complementary role for a stent—such as the BioMimics 3D—associated with durable outcomes is supported by the observation that while the pharmacokinetics of antiproliferative agents eluted from DCBs is time limited, loss of patency in the SFA due to restenosis occurs as late as 3 to 4 years after treatment [24]. DCBs alone do not provide the scaffolding afforded by a stent for overcoming the recoil and late negative remodeling that are contributory factors in the loss of patency. Besides providing such scaffolding, the BioMimics 3D has been shown in the MIMICS trial to be more capable than a straight nitinol stent in terms of significantly reducing the need for revascularization over an extended term of follow-up. It is thus anticipated that the combination of two different strategies for limiting NIH activation—the balloon delivery of antiproliferative drugs and the promotion of swirling flow leading to elevation of antirestenotic and atheroprotective wall shear—will have a synergistic effect on long-term clinical outcomes.

## Figures and Tables

**Table 1:** The MIMICS Clinical Trial Program.

Trial	MIMICS (NCT02163863)	MIMICS-2 (NCT02400905)	MIMICS 3D (NCT02900924)
Structure	Randomized controlled trial	Prospective registry, IDE study	Prospective registry, postmarket surveillance

Enrollment	Helical centerline arm (*n* = 50) Control stent arm (*n* = 26)	271 patients	Up to 500 real-world patients

Sites/location	8 sites/Germany	35 sites/United States 6 sites/German 6 sites/Japan	25 sites/Europe

Lesion type	Stenotic or occlusive lesions in the SFA; restenotic lesions permitted	Stenotic or occlusive lesions in the femoropopliteal artery	Stenotic or occlusive lesions in the femoropopliteal artery

Adjudication structure	Core labs: angiography, duplex ultrasound, X-ray	Core labs: angiography, duplex ultrasound, X-ray Independent clinical events committee	Independent clinical events committee

Primary endpoints	Objective performance goal efficacy and safety targets	Objective performance goal efficacy and safety targets	Efficacy: Freedom from CDTLR at 12 months Safety: Composite of MAE at 30 days

Secondary endpoints	Primary patency CDTLR Changes in Rutherford classification Changes in ABI measurements	Primary patency CDTLR Changes in Rutherford classification Changes in ABI measurements Changes in walking impairment questionnaire	Primary patency Changes in Rutherford classification Changes in ABI measurements

Subgroup analyses	Post hoc geometric analyses Presence and quantification of stented segment curvature based on geometric analysis of extended-leg and bent-knee X-ray measurements Presence and quantification of shear stress based on computational fluid dynamic modeling of duplex ultrasound data and bent-knee X-ray measurements	Presence and quantification of shear stress based on computational fluid dynamic modeling of duplex ultrasound data and bent-knee X-ray measurements	Adjunctive stenting with DCBs (*n* = 50) Popliteal lesions Calcified lesions

Inclusion criteria	Patients with Rutherford category 1 to 4 with symptoms considered due to SFA disease Lesion length: 4 cm to 10 cm, capable of being treated by a single stent	Patients with Rutherford category 2 to 4 due to PAD Lesion length: 4 cm to 14 cm capable of being treated by a single stent or by multiple stents	Patients with documented PAD who receive the helical centerline stent in accordance with the IFU

Exclusion criteria	Previous interventions at target site within 6 months Previous stent placement in target limb	Target lesion(s) requires percutaneous interventional treatment, beyond standard balloon angioplasty alone, prior to placement of the study stent	Patients whose lesions that cannot be crossed with a wire and/or balloon catheter and cannot be dilated sufficiently to allow passage of the delivery system

Follow-up	24 months	36 months	36 months

Status	Published results (Zeller et al. [25])	12-month results expected in 2018	12-month results expected in 2018

ABI, ankle/brachial index; CDTLR, clinically driven target lesion revascularization; DCB, drug-coated balloon; IDE, investigational device exemption; IFU, instructions for use; MAE, major adverse events; PAD, peripheral artery disease; SFA, superficial femoral artery; TASC, Trans-Atlantic Inter-Society Consensus.
